# More Hospital Beds for Civilian Patients?

**Published:** 1919-02-22

**Authors:** 


### MORE HOSPITAL BEDS FOR CIVILIAN PATIENTS?

In an article last week we referred to a rumour that seven members, out of a House Committee of thirty-five, exclusive of ex officio members, had summoned a meeting of the Governors of Westminster Hospital in the endeavour to throw over Sir Wolfe Barry's able scheme for a new hospital facing Clapham Common, with immediate accommodation for upwards of 300 beds. The Governors of Westminster Hospital duly met on the 18th inst., when a resolution was proposed and seconded by two of the Governors referred to which, if carried, would have committed the Governors to a policy of amalgamation with King's College Hospital, whilst preserving the name and traditions of Westminster Hospital. This was promptly met by an amendment to the effect that, before adopting a policy of amalgamation with King's College Hospital, an inquiry should be undertaken by two or more independent persons nominated by the Distribution Committee of the King's Fund to advise the Governors whether, in all the circumstances, it is expedient that Westminster Hospital and its medical school should be removed to the acquired site at Clapham or, in the alternative, that Westminster Hospital and its medical school should be amalgamated with some other similar institution or institutions. The amendment was carried unanimously, we understand.

As we stated last week an overwhelming majority of the active Governors of Westminster Hospital are credited with favouring the removal from Westminster to Clapham Common, where a hospital could supply the needs of some 40,000 people, and an excellent and popular medical school could be forthwith established. This large number of Londoners has long been in serious want of hospital accommodation and care. London is lamentably short of hospital beds for the protection and use of the civil population. Those who have the means to give largely to approved hospital schemes, and who take a deep, knowledgeable and intelligent interest in the future development of the hospital system of London, are practically unanimously in favour of the erection of the proposed hospital facing Clapham Common, as designed by Sir J. Wolfe Barry, by whose instructions the sketch plans were prepared of such a hospital and its surroundings. The two chief parties whose differences are imperilling the future hospital accommodation of thousands of Londoners are, first, those friends of King's College Hospital, which has over-spent its available funds for buildings, owing to the huge cost of those already erected, and the party of progress who desire to see Londoners provided with an adequate number of hospital beds to secure their safety and comfort as citizens of the metropolis of the Empire.

Those independent people who have weighed the list of men who have made themselves responsible for King's College Hospital have no doubt whatever that these gentlemen can, if they choose, readily raise any further money which may be needed for the completion of the hospital at Denmark Hill. If this be so the King's Fund will be placed in a most invidious position should it accept the invitation recently extended to it, to declare that Westminster Hospital shall be sunk in the Denmark Hill site, and the people of Clapham and the surrounding district deprived of the much needed hospital accommodation which Sir Wolfe Barry and his friends have generously placed at their disposal. The King's Fund was founded in 1897 with the object of securing more efficient aid and support for hospitals of London.

By confining itself to the purposes for which it was founded it has attained a position of authority and usefulness which is unique. For it to depart from the policy pursued in the past, for whatever reason, may win for it the resentment of poorer Londoners who may be deprived of the hospital beds they need, involving them in hardships and risks which would reflect no credit whatever upon those who may be sufficiently ill-judged to make such a cruel and unpopular policy possible. The proceedings of the King's Fund henceforth will have a very special interest for every intelligent Londoner, and we are confident that if the Fund's interests are represented by the Committee of Distribution, which possesses a unique knowledge of all the circumstances affecting hospital needs within the metropolis, poorer London may sleep in its bed by night, with full confidence that in the day of sickness it will not be deprived of hospital care through any action which may obtain the sanction and support of the Council of the King's Hospital Fund.

